# Evaluation of lung function in a German single center cohort of young patients with sickle cell disease using EIT and standard techniques

**DOI:** 10.3389/fmed.2023.1100180

**Published:** 2023-03-13

**Authors:** Alina Rein, Chuong Ngo, Maike van den Berg, Svenja Böll, Lisa Lassay, Udo Kontny, Norbert Wagner, Steffen Leonhardt, Klaus Tenbrock, Eva Verjans

**Affiliations:** ^1^Department of Pediatrics, Medical Faculty, RWTH Aachen University, University Hospital Aachen, Aachen, Germany; ^2^Medical Information Technology, Helmholtz Institute for Biomedical Engineering, RWTH Aachen University, Aachen, Germany

**Keywords:** sickle cell disease, lung function, electrical impedance tomography, sickle cell chronic lung disease, pediatric pulmology

## Abstract

**Background and objective:**

Sickle cell disease (SCD) is a very common autosomal recessive hemoglobinopathy leading to multiple pulmonary complications that are closely associated with mortality. The pathophysiology of chronic pulmonary involvement is not yet fully understood and no specific therapies are available.

**Methods:**

The aim of this cross-sectional study was to characterize the lung function of children and young adolescents with SCD in a German single-center cohort and to extend conventional lung function testing by the use of a new imaging method. We performed spirometry and body plethysmography in 35 children and young adults with hemoglobin SS, SC, S/β-thalassemia as well as 50 controls. These data were compared with clinical characteristics and typical laboratory parameters of hemolysis and disease activity in SCD. To identify lung inhomogeneities, for example due to atelectasis, hyperinflation, air trapping or vascular occlusions, we used the promising new method of electrical impedance tomography (EIT) and calculated global inhomogeneity indices.

**Results:**

Lung function of patients with SCD was significantly reduced compared to that of healthy controls. When the result was found to be pathological, the most commonly observed type of breathing disorder was classified as restrictive. Laboratory parameters showed typical features of SCD including decreased levels of hemoglobin and hematocrit and elevated levels of leucocytes, platelets, lactate dehydrogenase and total bilirubin. However, there was no correlation between blood values and reduced lung function. Electrical impedance tomography (EIT) revealed no abnormalities in SCD patients compared to healthy controls. In particular, we were unable to demonstrate any regional inhomogeneities in lung ventilation.

**Conclusion:**

In our study, SCD patients showed impaired lung function, with a relevant percentage of patients suffering from restrictive breathing disorder. Signs of obstruction could not be detected. Electrical impedance tomography (EIT) measurements revealed no unevenness that would suggest air entrapment, blockage of blood vessels, excessive inflation, obstruction, or other forms of lung disease. Additionally, the reduction in lung function observed in SCD patients was not related to the disease severity or laboratory test results.

## Introduction

Sickle cell disease (SCD) is a common autosomal recessive hemoglobinopathy, which can lead to a dramatic reduction in quality of life and average life expectancy. As one of the most common monogenetic disorders globally, it affects nearly 1 in 600 African Americans, up to 4% of children in sub-Saharan Africa being born with this disorder (1). The number of individuals affected in Germany is around 3,000 and rising, approximately 200 children being born with this hemoglobinopathy each year (2, 3).

SCD is caused by a point mutation in the gene encoding the ß-globin chain of hemoglobin. In stressful situations, such as infections and other hypoxia-inducing diseases, red blood cells deform into a sickle shape (4). These malformed erythrocytes can clot together and cause a vascular occlusion crisis characterized by hemolysis and vascular damage which may result in acute and chronic organ damage (5).

*“Sickle cell acute lung disease,”* the acute thoracic syndrome (ATS), is associated with the highest mortality of all disease-related complications and is one of the most common causes of hospitalization (6–9). The pathophysiology of the chronic form of lung involvement, known as *“sickle cell chronic lung disease,”* is still not fully understood, and no specific therapeutic options are yet available (10).

We therefore conducted and evaluated the present study to better characterize the lung function in pediatric patients with sickle cell disease treated at our center and to extend pulmonary diagnostics using a new imaging technique as a pilot study. For this we used electrical impedance tomography (EIT), a noninvasive radiation-free examination tool. In previous pediatric studies, EIT has been successfully applied to children with cystic fibrosis (11, 12) and bronchial asthma (13). The EIT-based global inhomogeneity (GI)-index is an especially promising tool for measurement of ventilation heterogeneities (14), which could be detectable in case of air trapping, vascular occlusion, hyperinflation or obstructive lung disease. However, up to now, EIT has not been performed on children with sickle cell disease.

## Materials and methods

### Population and study protocol

This clinical-experimental study was monocentric, prospective and open-labeled. It was approved by the Ethics Committee of the RWTH Aachen (EK234/19) and written informed consent was obtained from all children and/or their legal guardians before data acquisition. The study population that underwent pulmonary function testing consisted of pediatric patients and young adults with sickle cell disease (*n* = 35), and mostly age-matched healthy controls without any inflammatory or lung disease (*n* = 50). Both study groups were recruited at the department of pediatrics at RWTH Aachen University Hospital, Germany. Sickle cell patients were patients from the children’s hematooncology sickle cell outpatient clinic who came for routine check-ups. They did not suffer from acute sickle cell crisis. In total, this consultation has about 50 patients, about 40 of them are at least five and maximum 23 years old. Thirty-five of them participated in our study. Some of the others underwent stem cell transplantation and one did not want to participate. Inclusion criteria were an age of 5 to 23 years for both groups and, for the SCD group, genotype SC, SS or S-ß-Thal. Exclusion criteria were any systemic disease that could affect lung function in the control group and completed stem cell transplantation in the SCD group. Controls were recruited from the hospital inpatient or outpatient clinic and did not suffer from any acute or chronic lung disease. We took body measurements in both groups and medical history, including the number of pain crises per year and acute thoracic syndromes (ATS) per lifetime, and other sickle cell disease-related complications in the SCD group. We then calculated a self-developed organ score which describes the number of organ systems chronically affected by the disease. The organ score was designed based on the German guidelines for sickle cell disease (15). This scoring system provides a simplified overview over the complication rate of the individual patient. The score has up to know no documented prognostic value. Inclusion criteria were chronically increased flow velocity in the carotid Doppler, stroke and other cerebrovascular disease for complications of the brain. For the cardiac and pulmonary system, criteria were ventricular hypertrophy, hypertrophic cardiomyopathy, myocardial infarction, pulmonary hypertension and acute thoracic syndrome. The major ophthalmologic complication is proliferative retinopathy. Hepatobiliary disease included liver sequestration crisis, intrahepatic cholestasis and cholecystitis/cholelithiasis as well as girdle syndrome. Chronic renal failure, glomerulopathies and recurrent priapism were inclusion criteria for the renal and urinary system. Complications of the spleen were splenic sequestration crisis, splenic infarctions and splenectomy. Skeletal complications were osteomyelitis, bone necrosis and arthritis. Each category was rated with maximum one point when one of the mentioned complications has been previously diagnosed. The maximum organ score was 8, the lowest score without complications during lifetime was 0.

We further developed a pain crisis score, which determines the pain crises per year that required inpatient treatment. In contrast, the acute thoracic system score refers to the number of ATS in a patient’s lifetime and is therefore not averaged over age.

We also analyzed laboratory parameters containing hemoglobin (Hb), hematocrit (Hct), leukocyte count, platelet count, lactate dehydrogenase (LDH) and total bilirubin.

Blood tests were available from 34 of 35 SCD patients as it belongs to their routine checkup once a year. In the control group, blood was drawn from 30 out of 50 patients, depending on the purpose of their appointment in our hospital. Therefore, all presented blood parameters were determined at time of lung function testing during the last 12 month.

Spirometry and body plethysmography were performed with MasterLab, Care Fusion, Hoechberg, Germany. Spirometry was performed according to American Thoracic Society and European Thoracic society guidelines (16). As our control group was homogenously composed of European individuals of white race, and the SCD patients were of African black race, we corrected the spirometric measurements based on race using the GLI-2012 formula.

EIT measurements were performed with a PulmoVista 500 (Draeger, Lübeck, Germany), using the corresponding electrode belts of size XXS-L, placed two centimeters below the mammillary region. After a full body plethysmography, EIT and spirometry were performed simultaneously, all taken in a sitting position.

### EIT measurements

Electrical Impedance Tomography (EIT) is a noninvasive method for monitoring impedance changes in a cross section of body tissues (here: thorax) spanned by an electrode arrangement (here: a belt with 16 electrodes) around it (17). A harmless alternating current (IEC60601-1-2, 80–130 kHz, with automatic adjustment of current amplitude) is injected and the resulting voltages are measured by 16 consecutive current injections and 13 adjacent electrode pairs, which makes a total of 208 voltages during each cycle (Supplementary Figures S1, S2). Then the electrode pair for current injection is rotated, and the procedure repeated. Finally 16 × 13 = 208 voltage measurements are obtained which are subsequently used for impedance image reconstruction. An integrated finite-element-based Newton–Raphson reconstruction algorithm is applied to create a 32×32 image of the relative impedance distribution (18). We note that summing all pixels of this image provides the global relative impedance


$$\Delta \mathrm{Zglobal}\;\left(t\right)=\sum \Delta \mathrm{Zi}\left(t\right)$$

which is known to strongly correlate with tidal volume. After computing an image (a frame), the procedure is repeated from the beginning. In most current devices, the resulting frame rate is 40–50 frames per second (fps) indicating a high temporal resolution, while the spatial resolution is limited to 10–20% of the diameter (for the thorax, *ca.* 2–3 cm).

EIT has proven to be capable of monitoring regional ventilation by looking at the individual 𝛥Zi(t) pixels or groups of pixels [like layers, quadrants, etc., see (19)]. Meanwhile, there exist several metrics to quantify regional lung ventilation properties, including, e.g., the center of ventilation (20–22), the regional ventilation delay (23, 24), regional time constants (25), and the global inhomogenity index (26).

### Determination of the global inhomogeneity index

To determine the global inhomogeneity index from raw data, forced inspiration (FI) curves were manually extracted and flow volume (FV) loops were computed. The reconstruction of the EIT-related FV loops of the flow volume loops has been reported in our previous work (13). By comparing FV loops obtained by EIT to loops obtained by spirometry, the most accurate FI curve was used to determine the GI index in accordance with Zhao et al. (14, 26).

For this, the lung area was identified using the functional EIT method described in the publication of Hahn et al. (27). It is assumed that a pixel belongs to the lung area if the variation in pixel values of the EIT images during a certain period is larger than a threshold of 20% of the maximum variation. This lung area was then defined more closely using the “*Lung Area Estimation”* method (28). In this step, a heart frequency of 80 bpm was assumed for all patients. After determining the lung area, the GI index could be computed according to the algorithm presented (26). Thus, difference images representing the differences of impedance between 25, 50, 75, 100% of maximum inspiratory volume (maximum impedance) and the start of inspiration (minimum impedance) were evaluated using the following equation:


$$GI=\frac{\sum_{x,y\in lung}\left|DI_{xy}-Median\left(DI_{lung}\right)\right|}{\sum_{x,y\in lung}DI_{xy}}$$

The method resulted in four GI index values at the levels of 25, 50, 75 and 100% defined as GI_25_, GI_50_, GI_75_, and GI_100_, respectively.

### Computation of EIT images

Difference images were computed to illustrate the results. The raw data represent 32 × 32 pixel images over the course of the examination. To obtain difference images, pixel values of the image at the end of forced expiration were subtracted from the pixel values at the maximum inspiratory volume. Pixels were arranged in a 32 × 32 image with the color scale set as follows: the minimum value was set to 75% of the minimum value of the difference image and the maximum value was set to 75% of the maximum value of the difference image. Although some accuracy is lost, this allows for a clearer difference image that illustrates the regional ventilation of the lung.

### Statistical analysis

Quantitative results are expressed as means ± standard deviation (SD). Statistical analysis was performed with a general linear mixed model (GLMM) (PROC GLIMMIX, SAS9.4, SAS Institute Inc., Cary, United States), assuming a normal distribution except for cell counts (e.g., platelets), which are distributed negatively binomially. Homoscedasticity was tested using the covtest statement. In case of heteroscedasticity of the data, degrees of freedom were adjusted using the Kenward-Roger approximation. Multiple comparisons were corrected using the Shaffer-simulated (SIM) stepdown procedure. A *p* value of <0.05 was considered statistically significant (* *p* < 0.05, ** *p* < 0.01, *** *p* < 0.001).

## Results

### Characterization of the study population

The characterization of both groups of the study population is shown in Table 1. As mentioned above, we studied a cohort of 35 sickle cell patients (SCD) and 50 age-matched controls with no documented lung disease. Among the sickle cell patients, 77% were homozygous for HbS and 14% showed the SCD-S/C genotype. Additionally, 9% (3 patients) had an SCD-S ß-thalassemia genotype. The mean age was 15.4 (± 6.05) years in the sickle cell group and 14.06 (± 4.83) among the control patients without showing a significant difference between the two groups. Although the mean BMI of the sickle cell patients was lower (19.7) than that of the controls (20.9), there was no statistically significant difference. The entire SCD group was on hydroxyurea treatment, as it is standard practice in our clinic from age five onwards. Patients in the control group did not take any blood count-altering medication or medication for bronchodilatation. Six of our 35 SCD patients participated in a chronic transfusion program. While the study was running, none of the SCD patients took asthmatic medication like inhalative steroids or ß2-sympathomimetica. Six of them took asthmatic medication in the past with only limited success and no clinical history of asthma.

**Table 1:** Clinical characteristics of SCD patients and controls.

	SCD	Controls	*p*-Value
Number of patients	35	50	
Demographics and genetics			
Age (mean ± SD)	15.4 (± 6.05)	14.06 (± 4.83)	0.4480
Gender (n,(%))			
Male	23 (66)	21 (42)	0.0752
Female	12 (34)	29 (58)	0.2610
Genotype (%)			
SCD-S/S	27 (77)		
SCD-S/C	5 (14)		
SCD-S/ß-Thal	2 (6)		
Physical examination			
BMI (kg/m^2^, mean ± SD)	19.7 (± 4.97)	20.9 (± 4.03)	0.0692
Weight (kg, mean ± SD)	49.4 (± 21.38)	54.9 (± 19.76)	0.2315
Height (m, mean ± SD)	1.55 (± 0.22)	1.58 (± 0.19)	0.3949

### Impaired forced expiratory volume during the first-second (FEV1) and normal FEV1/FVC ratio in patients with sickle cell disease in our cohort

In our German cohort, we initially analyzed the FEV1 of sickle cell patients and controls using spirometry that was adjusted for ethnicity.

**Figure 1:**
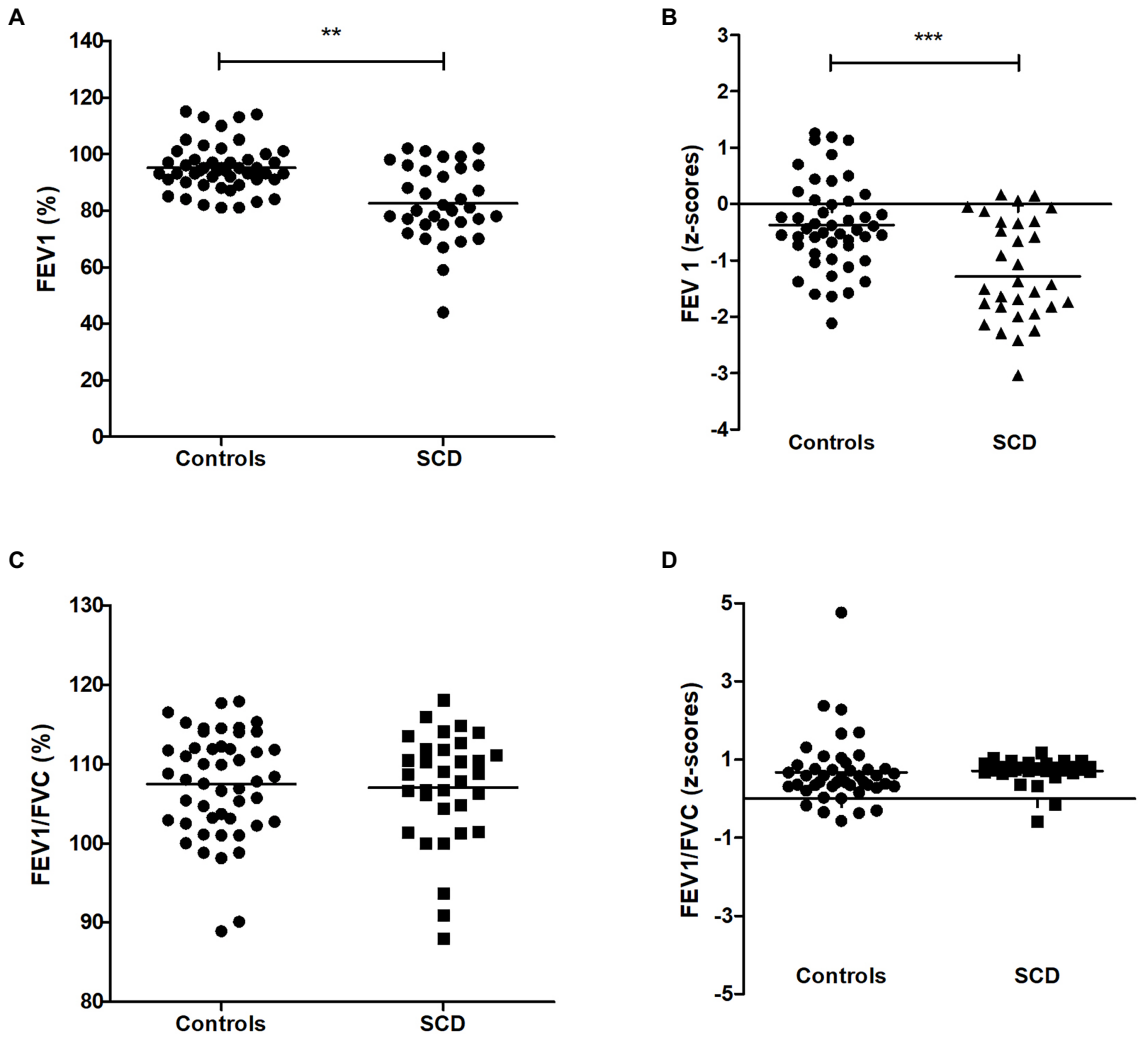
FEV1 of SCD patients and controls **(A)** Percent predicted forced expiratory volume during the first-second (FEV1 in %), **(B)** FEV1 (z-scores), **(C)** FEV1/FVC ratio, **(D)** FEV1/FVC ratio (z-scores). *** *p* < 0.001, ** *p* < 0.05.

The percent predicted FEV1 was lower among sickle cell patients compared to the control group (Figure 1A). As a result, the corresponding z-scores of the SCD group were also reduced (Figure 1B). 43% of the SCD patients in our study had pathological FEV1 levels, as indicated by their FEV1 percentage being below 80% and their z-scores being lower than the lower limit of normal (−1.65), as shown in Table 2. In contrast, only 2 of the control patients had FEV1 levels that were below the lower limit of normal (LLN).

**Table 2:** Lung function parameters in percentage values and z-scores.

	SCD	Controls	*p*-Value
Lung function parameters in % (mean, SD)			
FEV1	82.55 ± 13.33	95.14 ± 8.45	0.0013
FVC	76.04 ± 12.79	88 ± 10	0.0003
FEV1/FVC	107.0 ± 6.98	107.5 ± 6.61	0.8686
FEF 25–75	103.9 ± 26.09	105.1 ± 20.82	0.8999
TLC	76.71 ± 10.68	98.4 ± 14.20	0.0001
RV	98.45 ± 42.04	116.15 ± 37.74	0.0143
RV/TLC	1.21 ± 0.47	1.15 ± 0.35	0.3760
Lung function parameters (z-scores)			
FEV1	−1.28 ± 1.02	−0.37 ± 0.11	0.0006
FVC	−1.69 ± 1.04	−0.88 ± 0.13	0.0004
FEV1/FVC	0.70 ± 0.33	0.67 ± 0.87	0.0221
FEF 25–75	0.0977 ± 0.92	0.42 ± 0.94	0.1732
TLC	−2.82 ± 0.28	−0.08 ± 0.28	<0.0001
RV	−0.14 ± 0.36	0.97 ± 0.35	0.0406
RV/TLC	0.144 ± 0.42	0.504 ± 1.99	0.6504

**Figure 2:**
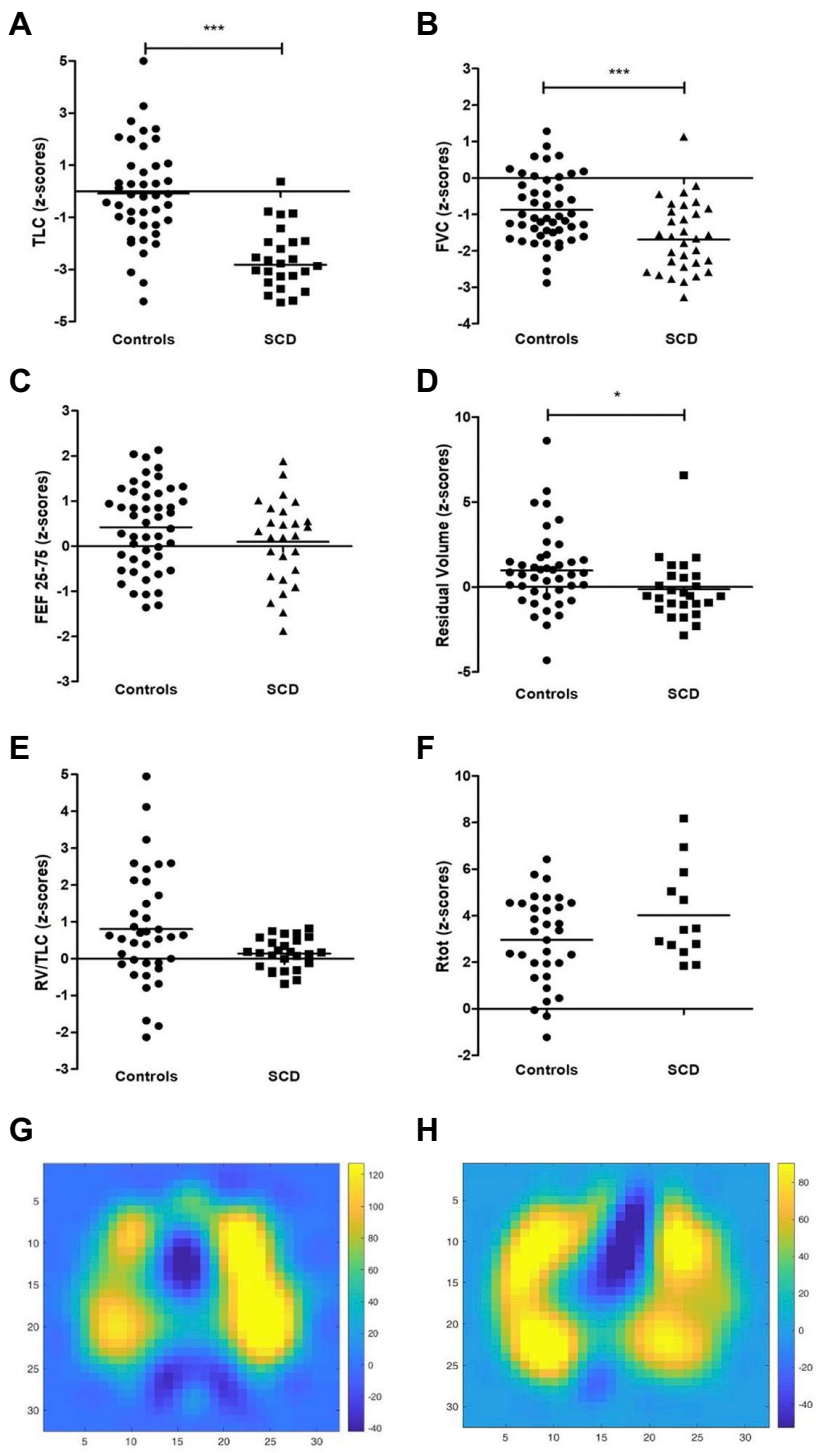
Additional lung function parameters of SCD patients and controls and examples of EIT difference images **(A)** TLC (z-scores), **(B)** FVC (z-scores), **(C)** FEF25-75 (z-scores), **(D)** RV (z-scores), **(E)** RV/TLC (z-scores). **(F)** Rtot (z-scores), **(G)** difference image of control patient and **(H)** SCD patient at forced expiration in 32 × 32 pixel images over the course of the examination with the color scale set as follows: the minimum value was set to 75% of the minimum value of the difference image and the maximum value was set to 75% of the maximum value of the difference image. Yellow color indicates higher differences and thus more ventilation.

**Figure 3:**
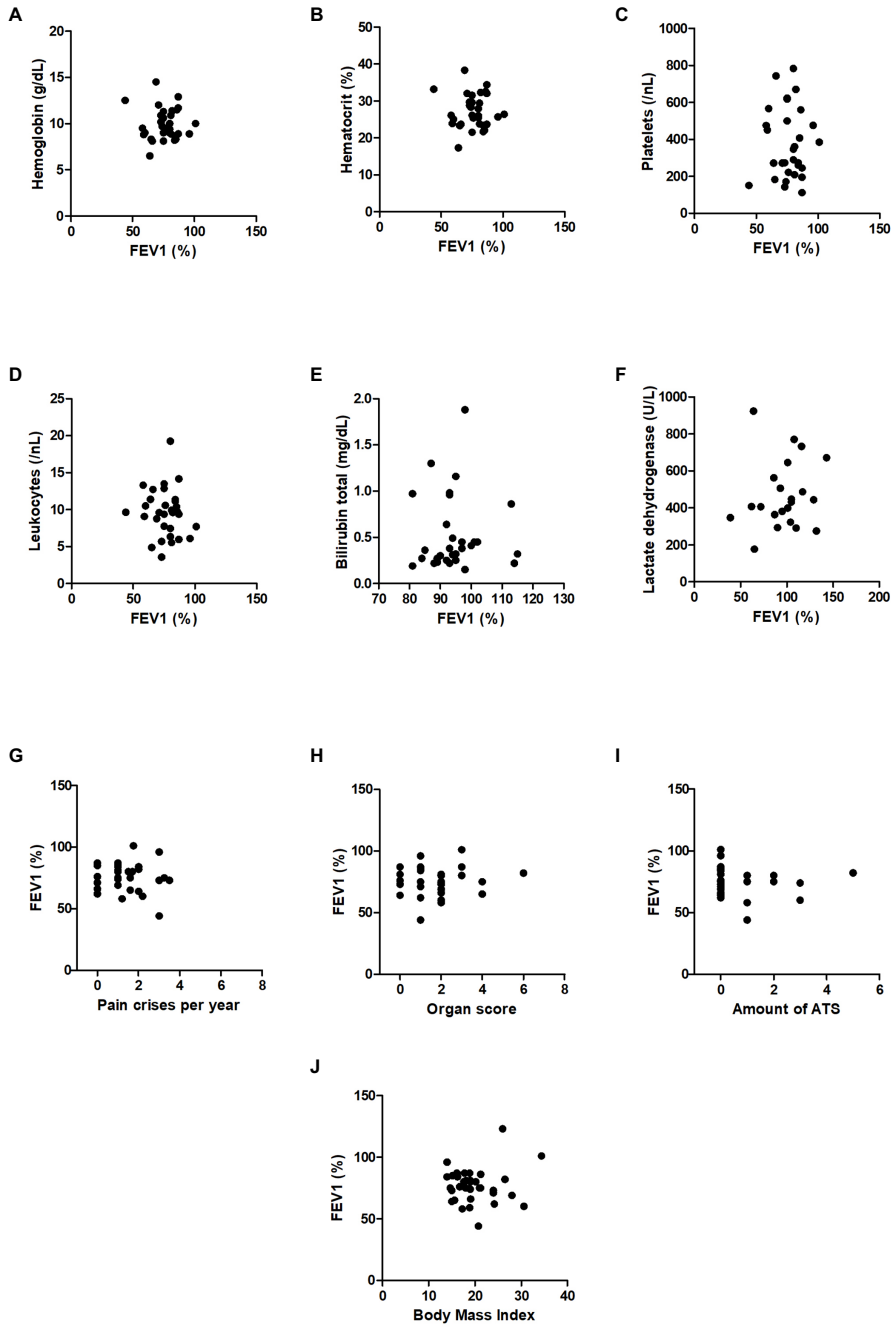
Correlations of blood parameters with FEV1 and correlations of FEV1 with disease activity parameters in SCD patients. **(A)** Serum levels of hemoglobin (g/dL), **(B)** hematocrit (%), **(C)** platelets (/nL), **(D)** leucocytes (/nL), **(E)** total bilirubin (mg/dL), **(F)** lactate dehydrogenase (U/L), **(G)** pain crises per year, **(H)** organ score, **(I)** amount of ATS, **(J)** body mass index.

At the same time, the FEV1/FVC ratio, indicating obstructive components, was normal in both study groups and revealed no significant differences [(Figure 1C) and z-scores in (Figure 1D)], while FVC was also reduced in the SCD group (z-scores in Figure 2B, other values in Table 2). To correlate pulmonary function with laboratory parameters, we analyzed the blood values of SCD patients and controls. Patients with sickle cell disease did indeed show typical hematologic features including significantly lower levels of hemoglobin, hematocrit and higher levels of leukocytes, platelets, lactate dehydrogenase and total bilirubin compared to control patients (Table 3). However, when we correlated these blood parameters with FEV1 and FEV1/FVC ratio in the SCD group, we could not find any relevant correlations (example given in Figures 3A–F), with other correlations and z-scores supplied in Supplementary Figures S2, S3A–F. Thus, hematological features were not associated with a reduction in lung function in our single-center cohort.

**Table 3:** Hematological characteristics of SCD patients and controls.

	SCD	Controls	*p*-Value
Blood parameter (mean ± SD)			
Hemoglobin (g/dL)	9.87 ± 1.67	13.27 ± 1.21	0.0002
Hematocrit (%)	27.04 ± 4.51	38.91 ± 3.63	<0.0001
Leukocyte count (/nL)	9.26 ± 3.41	6.87 ± 2.19	0.0019
Platelet count (/nL)	356.79 ± 192.66	278.82 ± 84.81	0.0383
Lactate dehydrogenase (U/L)	458 ± 185	205 ± 55	<0.0001
Total bilirubin (mg/dL)	2.31 ± 1.23	0.54 ± 0.40	<0.0001

Results expressed as median and standard deviation.

Next, we correlated FEV1 and FEV1/FVC-ratio of SCD patients with markers of disease activity such as the organ score, the pain crisis score and the number of ATS in lifetime (example given in Figures 3G–J), with other correlations and z-scores supplied in Supplementary Figures S2, 3G–J. In summary, we did not observe any correlations between FEV1 or FEV1/FVC-ratio and disease activity scores. Thus, disease activity did not correlate with lung function impairment in the group of our SCD patients studied.

### Restriction is the predominant finding in SC lung disease in our cohort

Thereafter, we investigated additional lung function parameters *via* body plethysmography. Besides reduced FEV1 and normal FEV1/FVC-ratio values, several SCD patients showed further signs of restriction compared to controls with normal lung function. A total of 60% of the SCD patients and 18% of the control patients had total lung capacity (TLC) values that were significantly reduced to pathological levels below LLN, as demonstrated by their z-scores in Figure 2B and Table 2, and additional values in the Supplementary Figure S4.

Additionally, there were no differences in airway resistance or FEF25-75 between the two groups, and the residual volume was even lower in the SCD group (z-scores in Figures 2C,D and Table 2, other values in Supplementary Figure S4). Therefore, our study revealed well-established characteristics of restriction, including reduced FEV1, normal levels of FEV1/forced vital capacity (FVC), reduced FVC, and reduced TLC, without any signs of an obstructive breathing disorder in our cohort. Furthermore, none of the lung function parameters in the SCD group were found to be associated with disease activity scores (data not shown).

To further investigate ventilation of SCD patients in our single-center cohort, we used the novel method of electrical impedance tomography. This method provides a means of locating inhomogeneities of the lung ventilation. For example, in the case of obstructive lung disease, we would expect to find both well-ventilated areas and areas with low ventilation or air trapping. Figure 2 exemplarily shows difference images of a control (G) and a SCD patient (H) calculated as following: pixel values of the image at the start of inspiration were subtracted from the pixel values at the maximum inspiratory volume during forced inspiration. Pixels were arranged in a 32 × 32 image with the color scale set as follows: The minimum value was set to 75% of the minimum value of the difference image and the maximum value was set to 75% of the maximum value of the difference image. Thus, the figures only present exemplarily pictures of one difference calculation.

Additionally, we calculated global homogeneity indices (GI) to detect overall lung inhomogeneities. Four difference images were calculated representing the differences of impedance between 25, 50, 75, 100% of maximum inspiratory volume (maximum impedance) and the start of inspiration (minimum impedance). Inhomogeneity indices were calculated over the whole lung and including all four previous calculations. Altogether, we could not find any differences between the two groups (Table 4). Thus, these experiments underlined the absence of ventilation inhomogeneities in our SCD cohort.

**Table 4:** Global inhomogeneity index of SCD patients (*n* = 16) and controls (*n* = 26) at the level of 25, 50, 75 and 100% inspiratory volume.

	SCD	Controls	*p*-Value
Global inhomogeneity index (mean ± SD)			
GI 25	0.58 ± 0.04	0.6 ± 0.07	0.2542
GI 50	0.55 ± 0.06	0.57 ± 0.07	0.4377
GI 75	0.53 ± 0.07	0.54 ± 0.07	0.9166
GI 100	0.52 ± 0.08	0.52 ± 0.08	0.9400

## Discussion

Our findings clearly demonstrate that lung function is compromised in a relevant number of young SCD patients in our single-center cohort.

These alterations in lung function did not correspond to changes in blood parameters or disease activity, including different disease activity scores. The predominant pattern of lung dysfunction in our pediatric SCD cohort was restrictive, and this was supported by the results of electrical impedance tomography (EIT) measurements, which showed homogeneity in lung ventilation.

Previous studies have been contradictory with regard to the predominant type of ventilatory disorder in SCD patients. Data from the PUSH study, which examined the lung function of children and adolescents in the U.S., showed most participants to have an obstructive ventilation disorder (29). Koumbourlis et al. detected normal patterns of lung function in 57% of the patients, lower airway obstruction in 35%, and restrictive lung disease only in 8% (30). In our study, typical features of restriction, such as reduced FEV1 with normal FEV1/FVC-ratio values were found in 43% of all patients but in general, all patients tended to have lower FEV1 levels compared to controls, even when these values were still in non-pathological ranges. Furthermore, FVC, TLC and RV were significantly and mostly even pathologically reduced compared to controls, while FEF 25–75 and total resistance did not differ between both groups. Thus, our results match those of Sylvestre et al., who reported that restrictive ventilatory disorder appears in SCD children (31). However, the mean age of our study participants was 4 years higher than that of Sylvestre’s study population. Furthermore, we performed total body plethysmography with most patients, which is considered indispensable for the diagnosis of restriction. Since we performed a cross-sectional study, no statement can be made about the longitudinal changes of lung function. We also diagnosed single features of obstructive lung disease in some of our SCD patients (*n* = 6). However, these patients did not respond to therapy with inhaled corticosteroids and had no clinical signs of asthma. Thus, we stopped anti-obstructive treatment months/year before.

In line with our data, Lunt et al. demonstrated that the majority of adults with SCD suffered from restrictive ventilatory disorders (32, 33). They reported a correlation between the reduced vascular volume of the lungs and extracellular matrix proliferation, potentially leading to restriction. Stauffer et al. came to a similar conclusion: they examined a group of homozygous adult sickle cell patients and found restrictive ventilatory disorder in 32% of the subjects. These patients also had a significantly reduced diffusion capacity, which they attributed to a reduced alveolar volume (34).

Using electrical impedance tomography (EIT), we tried to further characterize impaired lung function in our SCD cohort. EIT offers the chance to identify inhomogeneities in lung ventilation, for example as an early indication of local hyperinflation and air trapping caused by well-ventilated areas lying next to areas with restricted ventilation (35). We have used this technique before to identify inhomogeneities in patients with cystic fibrosis and asthma (11–13, 36). In these studies, EIT measurements showed strong correlation with common pulmonary function testing. Even positive bronchospasmolysis was detectable *via* EIT. In our cohort, we could not find any inhomogeneities in the lung ventilation of SCD patients. As a consequence, it may be concluded that our patients did not suffer from atelectasis, hyperinflation, vascular occlusion, severe obstruction, or other causes of inhomogeneous ventilation. Other reasons for the absence of ventilation inhomogeneities may be erroneous or non-sensitive measurements. Since we have checked all measurements for sufficient quality and obstructions have been successfully measured *via* EIT in the past, we consider these possibilities to be unlikely. However, it can be assumed that minor inhomogeneities are not (yet) detectable.

Besides the type of ventilatory disorder, the pathophysiological mechanisms underlying these restrictive patterns are not fully understood. Intuitively, one would expect repeated episodes of parenchymal injury to result in the deposition of fibrotic tissue, leading to restrictive lung disease. Some people assume that ATS develops into the chronic form (sickle cell chronic lung disease, SCCLD), resulting in irreversible lung parenchyma damage (37). However, Knight et al. described cases of SCCLD that were not due to previous episodes of ATS (38). The pulmonary restriction he observed did not correlate with the existence or number of ATS or indeed with any other manifest end-organ complications. It remains unclear how subclinical changes of SCD affect organs such as the lung. Endothelial dysfunction, sterile inflammation and impaired biorheology are hallmarks of SCD resulting in vaso-occlusion and end-organ injury (5, 39). We postulate that sterile inflammation, including the release of TNFα, IL-1β, IL-18 and IL-6, leads to changes in the endothelial-epithelial barrier and induces chronic fibrosis. Additionally, vaso-occlusion may induce small lung insults with a ventilation-perfusion mismatch (Euler-Liljestrand mechanism) and promote activation of typical immune cells (40) leading to chronic inflammation and lung tissue damage. As a consequence, ventilatory inhomogeneities would occur and become detectable *via* EIT measurements. However, our findings in children and young adults appear to contradict this, since we have been unable to detect any inhomogeneities as yet. Perhaps, changes in ventilation homogeneity appear later in life after several episodes of vaso-occlusion. It would therefore be most interesting to perform EIT measurements on adult SCD patients and as longitudinal studies. Some studies in the past have described an association of blood parameters, such as number of leukocytes, thrombocytes and LDH, with impaired lung function (29, 41, 42), the underlying notion being that inflammatory and hemolysis parameters directly induce pulmonary disorder. Arteta et al., for example, found that ventilation disorders in SCD patients were associated with an increase in LDH (29). In contrast, in our study we could not show any correlation of parameters of hemolysis and inflammation with lung function measurements. Hence, the argument that inflammatory blood cells directly induce chronic pulmonary disorder in SCD now seems outdated regarding our cohort.

In our study, lung function appeared to be independent of laboratory findings in SCD patients and also unrelated to the number of pain crises, ATS and other organ complications. These results agree with data of Cohen et al., who also studied a childhood cohort (43).

Regarding treatment of pulmonary complications in SCD, there are unfortunately no specific therapies to date. Zahran et al. showed that hydroxyurea (HU) therapy reduces inflammatory activity, decreases neutrophils, and ultimately not only improves blood counts but also decreases the incidence of vaso-occlusive episodes (VOC) (44). Nevertheless, according to the American Thoracic Society, the impact of HU on patients’ pulmonary function remains largely unclear (42). A very recent study retrospectively investigated the development of lung function in children with and without HU therapy, and found an increase in FEV1 and FVC values in the treated group while the non-treated group showed a decrease in these values (45). Van Geyzel et al. showed that children treated with HU had higher oxygen saturation overnight (46). Since all participants in our study were taking HU, we could not evaluate the benefit of this therapy. Further research is needed to elucidate the pathomechanism underlying SCD lung disease in order to develop specific disease-modeling therapies.

The limitations of our study are that we were unable to obtain EIT data from all SCD patients and control participants. Despite this, we were able to include lung function parameters for the majority of patients, as spirometry and body plethysmography were always available and each measurement underwent quality control checks. As a result, any low-quality analysis could be easily replaced with new ones.

Unfortunately, only 16 out of 35 sickle cell patients and 26 out of 50 controls consented to the electrical impedance tomography (EIT) measurement. This was due to some patients declining the measurement despite its harmlessness, as well as technical difficulties during the post-measurement analysis of EIT images. Some values were not recorded correctly due to a defective electrode in the chest belt and were excluded from the final measurements. As the patients only visit the outpatient unit once a year, it was not possible to repeat the measurements during the study period. Despite these limitations, the results of the pilot study showed no inhomogeneities in EIT for the sickle cell patients and controls, leading us to hypothesize a tendency toward restrictive, non-obstructive lung abnormalities in sickle cell disease. Furthermore, due to the cross-sectional design of our study, we cannot draw any conclusions regarding the progression or evolution of pulmonary function impairment over time. Another limitation of our study is the diversity of sickle cell genotypes in our cohort. However, we were fortunate to include nearly all SCD patients aged 5 years and older treated in our outpatient department. A larger, multi-center study that compares a single genotype across multiple centers would be highly informative.

We have demonstrated that the pattern of lung dysfunction in our young SCD cohort is restrictive, as FEV1 and TLC were often pathologically reduced, whereas FEV1/FVC showed normal values. Chronic reduction of lung function seems to be independent of clinical and laboratory findings. Lung damage in ATS does not appear to be associated with chronic restrictive changes in lung function in childhood. Eventually, a full recovery of lung function after ATS is therefore possible. EIT measurements showed no differences between control patients and sickle cell patients.

In the future, it will be crucial to conduct further EIT studies on SCD patients and use EIT to visualize ATS. Additionally, comparative studies on lung parenchyma perfusion would provide valuable insights.

## Data availability statement

The original contributions presented in the study are included in the article/Supplementary material, further inquiries can be directed to the corresponding author.

## Ethics statement

The studies involving human participants were reviewed and approved by Ethics Committee of the RWTH Aachen (EK234/19). Written informed consent to participate in this study was provided by the participants’ legal guardian/next of kin.

## Author contributions

AR, CN, EV, and KT were involved in the study design and collection of data. AR, SB, MB, and EV were responsible for data analysis. AR was responsible for drafting the manuscript and data interpretation. All authors were involved in critical revision and approval of the manuscript.

## Conflict of interest

The authors declare that the research was conducted in the absence of any commercial or financial relationships that could be construed as a potential conflict of interest.

## Publisher’s note

All claims expressed in this article are solely those of the authors and do not necessarily represent those of their affiliated organizations, or those of the publisher, the editors and the reviewers. Any product that may be evaluated in this article, or claim that may be made by its manufacturer, is not guaranteed or endorsed by the publisher.
