# Corrigendum: Evaluation of lung function in a German single center cohort of young patients with sickle cell disease using EIT and standard techniques

**DOI:** 10.3389/fmed.2023.1210947

**Published:** 2023-06-01

**Authors:** Alina Rein, Chuong Ngo, Maike van den Berg, Svenja Böll, Lisa Lassay, Udo Kontny, Norbert Wagner, Steffen Leonhardt, Klaus Tenbrock, Eva Verjans

**Affiliations:** ^1^Department of Pediatrics, Medical Faculty, RWTH Aachen University, University Hospital Aachen, Aachen, Germany; ^2^Medical Information Technology, Helmholtz Institute for Biomedical Engineering, RWTH Aachen University, Aachen, Germany

**Keywords:** sickle cell disease, lung function, electrical impedance tomography, sickle cell chronic lung disease, pediatric pulmology

In the published article, there was an error in the **Author contributions**. Instead of “AR, CN, EV, KT were involved in the study design and collection of data. AR, SB, MB and EV were responsible for data analysis. All authors were involved in the data interpretation and in the drafting, critical revision and approval of the manuscript.” it should be “AR, CN, EV, and KT were involved in the study design and collection of data. AR, SB, MB, and EV were responsible for data analysis. AR was responsible for drafting the manuscript and data interpretation. All authors were involved in critical revision and approval of the manuscript.”

The authors apologize for this error and state that this does not change the scientific conclusions of the article in any way. The original article has been updated.

