# Supplementary material for: Evaluation of lung function in a German single center cohort of young patients with sickle cell disease using EIT and standard techniques
Source: Front Med (Lausanne). 2023 Mar 13;10:1100180. doi: 10.3389/fmed.2023.1100180 (PMC10040809; doi:10.3389/fmed.2023.1100180)

**"Evaluation of lung function in a German single center cohort of young patients with sickle cell disease using EIT and standard techniques"**

**Supplemental material**

**Figure 1: Exemplary sequence of current injection and voltage measurement in an EIT device.**


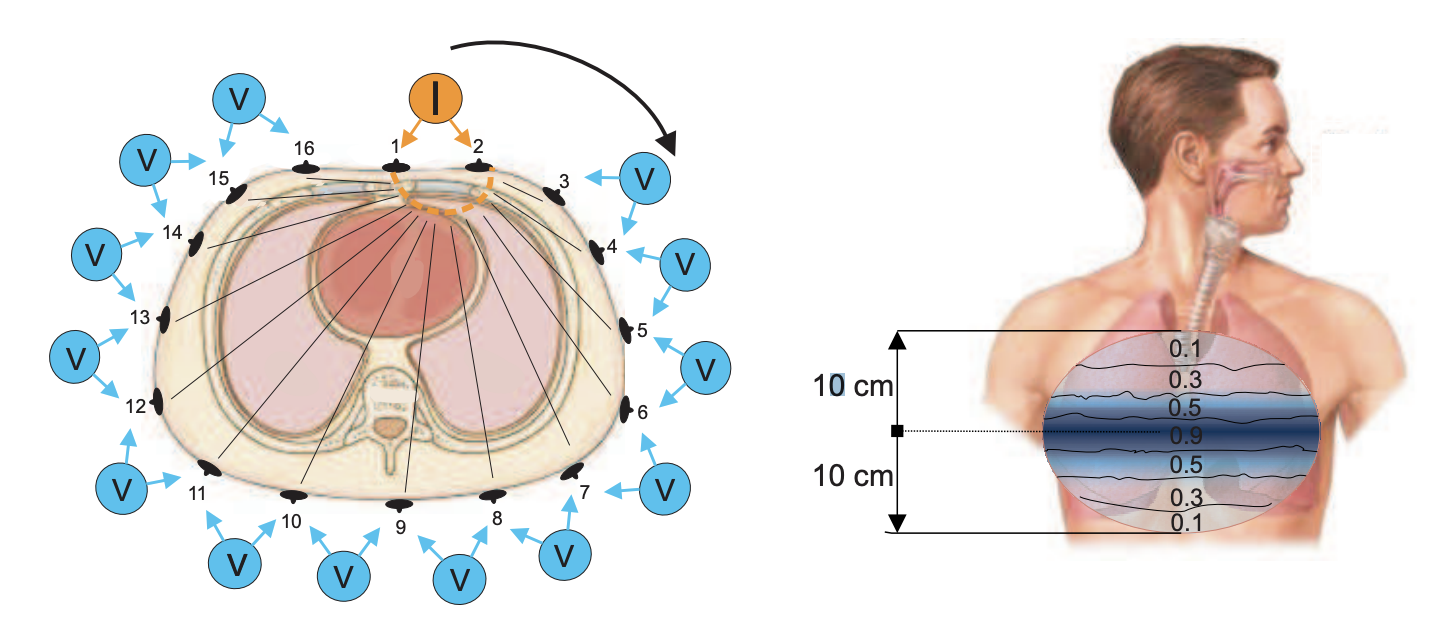


(Ngo Nguyen, “Model-based analysis of respiratory mechanics for diagnosis of cardiopulmonary diseases”, 2019, p. 32)

**Figure 2: Correlations of blood parameters and disease activity with FEV1/FVC ratio in SCD patients.**

Serum levels of **(A**) hemoglobin (g/dL), **(B)** hematocrit (%), **(C)** platelet count (/nL), **(D)** leucocyte count (/nl), **(E)** total bilirubin (mg/dL), **(F)** lactate dehydrogenase (U/l), pain crises per year **(G)**, organ score **(H)**, amount of ATS **(I)**, BMI **(J)**.


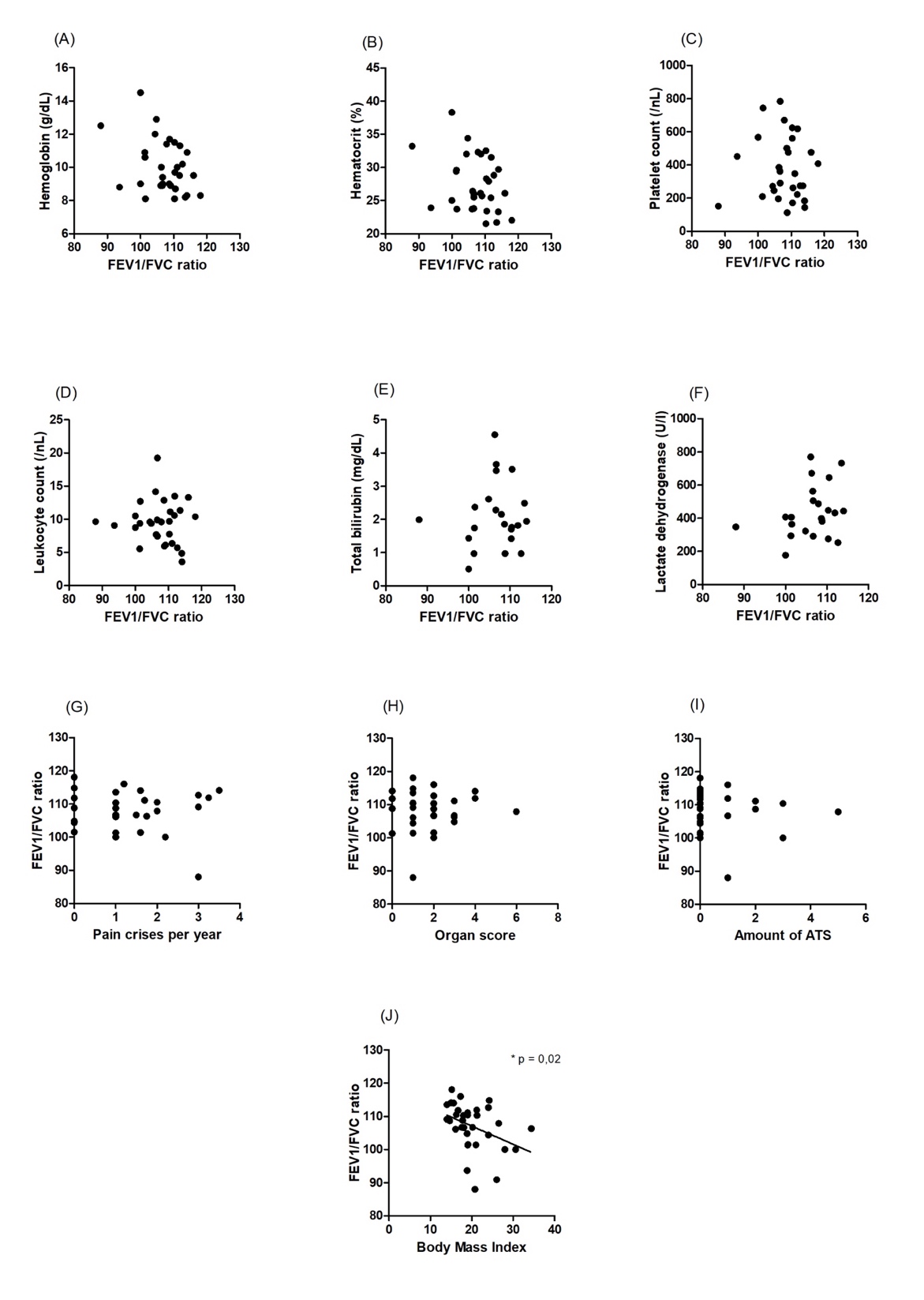


**Figure 3: Correlations of blood parameters and parameters of disease activity with z-scores of FEV1 in SCD patients.**

Serum levels of **(A**) hemoglobin (g/dL), **(B)** hematocrit (%), **(C)** platelet count (/nL), **(D)** leucocyte count (/nl), **(E)** total bilirubin (mg/dL), **(F)** lactate dehydrogenase (U/l), pain crises per year **(G)**, organ score **(H)**, amount of ATS **(I)**, BMI **(J)**.


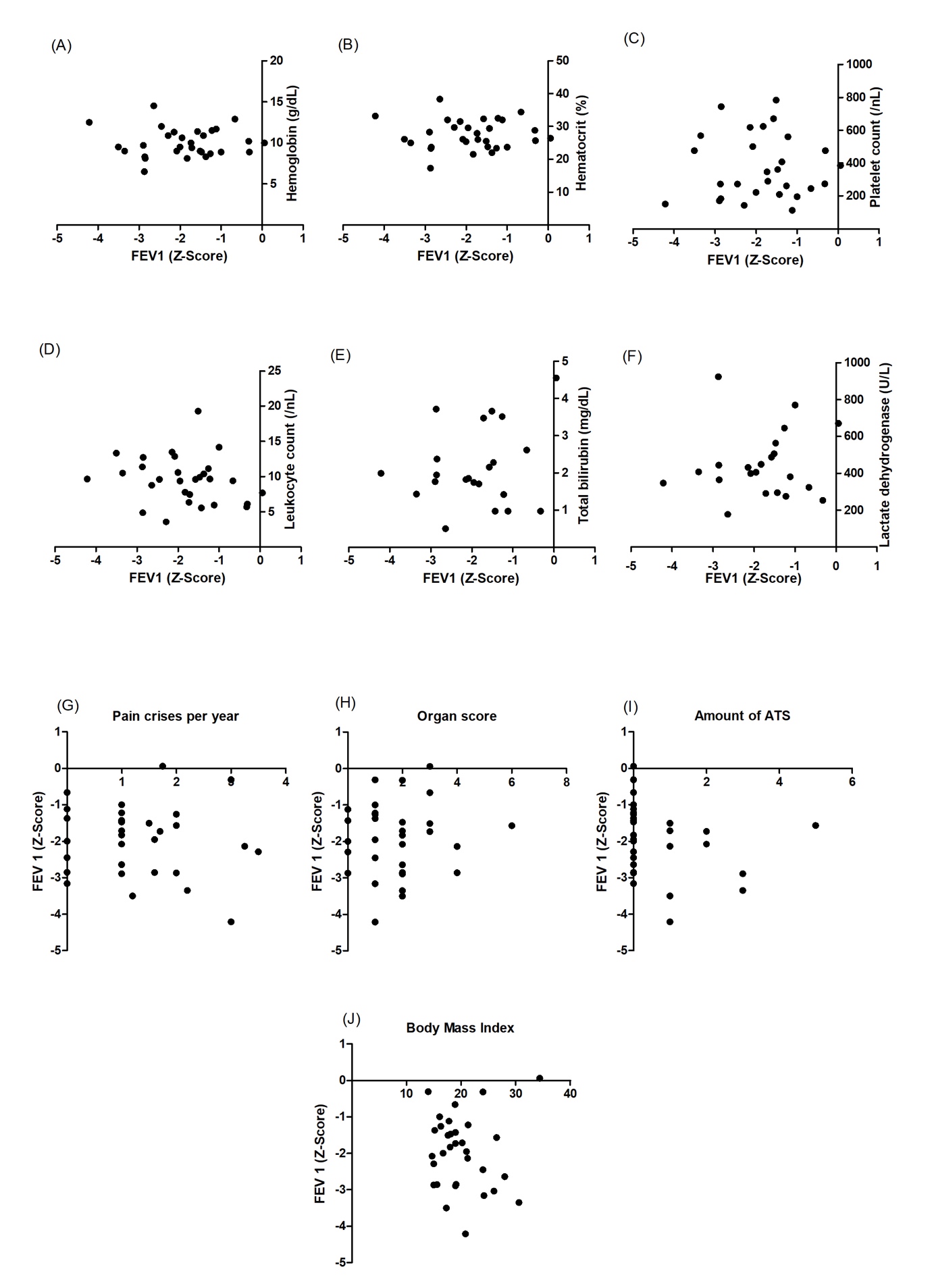


**Figure 4: Lung function of SCD cohort and controls**

(A) TLC, (B) FVC, (C) Rtot, (D) FEF25-75, (E) RV, (F) RV/TLC

*** p< 0.001, ** p< 0.05.


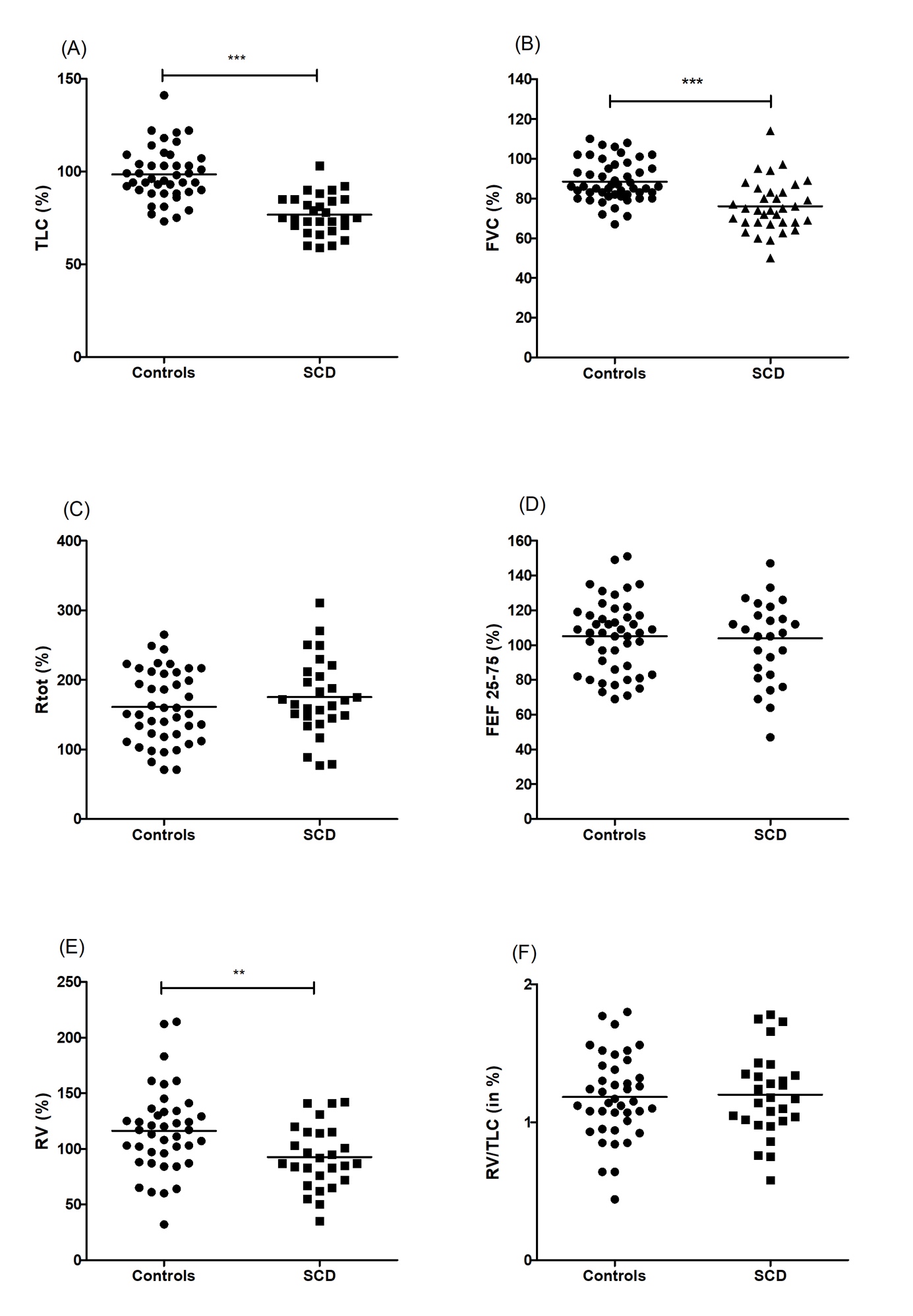

Supplement: Supplementary file 1 [file Data_Sheet_1.DOCX]
